# Personal

**Published:** 1888-10

**Authors:** 


### Personal.

Dr. Lucien Howe, of Buffalo, who went to Egypt last winter in order to study the bacillus of the ophthalmia of the East, has returned. Dr. Howe also incidentally investigated the leprosy of that country. He signaled his return to Buffalo by a most graceful act of courtesy to the profession, in an informal reception to Dr. Carl Koller, whose name is indissolubly associated throughout the world with the application of the anæsthetic properties of cocaine to surgery and medicine. About sixty guests assembled in the doctor's parlors at nine o'clock p. m., September 24th ult., and after an hour's conversation adjourned to the improvised dining-hall for two hours more of delightful intercourse. Speeches, songs by a detachment from the Liedertafel, and other forms of social intercourse, served to pass a most enjoyable evening.

---

DR. WM. A. HAMMOND, Surgeon-General U. S. A. (retired), was a conspicuous figure at the Congress of American Physicians and Surgeons in Washington last month. He will soon return to Washington to take up his residence after an absence of twenty-five years, where he will continue the practice of the specialty in which he has made such fame, having erected, near the city, a fine sanitarium for the accommodation of his patients.

---

DR. L. S. MCMURTRY, of Danville, was recently elected President of the Kentucky State Medical Society. This is a deserved recognition of the culture, erudition, and skill of one of Kentucky's most eminent physicians.

